# Improving Contraceptive Access, Use, and Method Mix by Task Sharing Implanon Insertion to Frontline Health Workers: The Experience of the Integrated Family Health Program in Ethiopia

**DOI:** 10.9745/GHSP-D-17-00215

**Published:** 2017-12-28

**Authors:** Yewondwossen Tilahun, Candace Lew, Bekele Belayihun, Kidest Lulu Hagos, Mengistu Asnake

**Affiliations:** a Pathfinder International Ethiopia, Addis Ababa, Ethiopia.; b Pathfinder International, Washington, DC, USA.

## Abstract

In 2009, the Ethiopian Federal Ministry of Health launched an Implanon scale-up program with the goal of improving the availability of long-acting reversible contraceptive (LARC) methods at the community level. The Integrated Family Health Program (IFHP) supported the ministry to train Health Extension Workers (HEWs), a cadre of frontline health workers, on Implanon insertion. Prior to this task-sharing initiative, HEWs were only permitted to provide short-acting contraceptive methods; Implanon insertion services were only available at higher-level health facilities, such as health centers and above. To train HEWs on Implanon insertion, IFHP followed a phase-based approach, which consisted of a learning phase (July to September 2009) that transitioned into a scale-up phase (December 2009 to December 2015). Training began with a series of service delivery-based training of trainers (TOT) sessions for clinical care providers selected from health centers followed by rollout trainings on Implanon insertion for HEWs selected from health posts. Immediately after the Implanon rollout trainings, each trained HEW was provided with consumables and Implanon implants to enable them to initiate the Implanon services at their respective health post. To reinforce knowledge and skills, we conducted mentoring visits and performance review meetings. From July 2009 to September 2015, 98 TOT sessions trained 2,328 clinicians and 320 rollout trainings reached 8,436 HEWs. A total of 1,382,318 women received contraceptive services through any IFHP-supported service delivery point, 1,273,990 of whom received an Implanon implant. The IFHP approach proved to be a successful model for increasing access to contraceptive methods in the community, and the program supported the integration of Implanon services into the existing public health service delivery system.

## Abstract (precis)

Between 2009 and 2015, 1.2 million women received Implanon implants from trained Health Extension Workers. Of the approximately 7,000 implant service visits made during the first 6 months, 25% were among women who had never used contraception before.

## INTRODUCTION

With an estimated 102 million people,^1^ Ethiopia has the second largest population in Africa. The average Ethiopian woman gives birth to 4.6 children in her reproductive years, and an estimated 22.3% of currently married women aged 15 to 49 have an unmet need for family planning.^2^ Modern contraceptive prevalence among all women has steadily increased over the last decade—from 9.7% in 2005^3^ to 18.7% in 2011^4^ and to 35.3% in 2016.^2^ Despite this increase, access to and use of long-acting reversible contraceptives (LARCs) remains limited in Ethiopia, with implants and intrauterine devices (IUDs) accounting for just 9.9% of total modern contraceptive use among all women in 2016 (7.9% and 2.0%, respectively).^2^

In Ethiopia, an estimated 22.3% of currently married women aged 15 to 49 have an unmet need for family planning.

LARCs offer highly effective protection from unintended pregnancy and contribute significantly to reducing unmet need for family planning.^5^ LARCs also prevent maternal and neonatal deaths by allowing women to delay childbearing, space births, avoid unintended pregnancy and abortion, and stop childbearing when they have reached their desired family size.^6^ LARCs are characterized by their effectiveness, length of efficacy, reversibility, and rapid and predictable return of fertility after discontinuation of the method. Implanon, a single rod subdermal implant that is inserted under the skin of the upper arm, prevents unintended pregnancy for at least 3 years. When removed, return to fertility is prompt.

In many countries, the severe shortage of skilled health care workers trained in contraceptive service provision is a key constraint to improving access to family planning services, including LARCs.^7^ Highly trained health care workers—typically stationed in health care facilities—often do not reach more marginalized populations, such as the unmarried, the young, the poor, migrants, and rural women. Task sharing, “the process of enabling lay and mid-level health care professionals—such as nurses, midwives, clinical officers and community health workers—to provide clinical tasks and procedures safely that would otherwise be restricted to higher-level cadres,”^8^ represents an important option for addressing the shortage and uneven distribution of health care workers.

In 2003, the government of Ethiopia launched the Health Extension Program, its flagship community-level health program, which created a new cadre of Health Extension Workers (HEWs) and shifted provision of certain health services to this all-female cadre. Women interested in becoming HEWs are eligible after they complete high school. They receive 1 year of training on 16 Health Extension Program packages, including family planning counseling and provision of short-acting contraceptive methods, specifically, combined oral contraceptive pills, progestin-only pills, progestin injectables, and condoms. The Ethiopian Federal Ministry of Health (FMOH) deploys 2 HEWs to each health post, the lowest level of the Primary Health Care Unit (PHCU). Each PHCU is composed of a health center and an average of 5 satellite health posts. The health center is responsible for providing administrative and technical support to the health posts.

In 2009, in order to increase the availability of LARCs at the community level, the FMOH asked organizations working in the health sector to pilot a task-sharing program to train HEWs to insert Implanon at health posts. After a successful pilot, the Integrated Family Health Program (IFHP)—implemented by Pathfinder International in partnership with John Snow, Inc. and funded by the United States Agency for International Development (USAID)—supported the FMOH to scale up provision of Implanon by HEWs.

In 2009, in order to increase the availability of LARCs at the community level, the FMOH asked organizations working in the health sector to pilot a task-sharing program to train HEWs to insert Implanon at health posts.

IFHP supports the FMOH to provide integrated family planning and maternal, newborn, and child health services and improve the quality of reproductive health services in public sector health facilities in Amhara; Oromia; the Southern Nations, Nationalities, and People's Region (SNNPR); and Tigray. The program supports and strengthens primary health care services provided by HEWs in their communities as well as services provided at health centers. The program is active in more than 300 *woredas* (districts) in these regions, supporting a total of 1,378 health centers and 6,378 health posts.

The primary aim of this article is to describe the process by which Implanon insertion services were shifted to HEWs in Ethiopia, so that implementers may use our experience to pursue task sharing of contraceptive service provision in their own settings. By identifying gaps in the program, exploring key programmatic findings, and evaluating results from service delivery data collected during program implementation, as well as a client survey, our aim is also to contribute to the evidence base on how best to increase access to a wide range of contraceptive methods.

## METHODS

### Program Implementation

#### Pre-Program Implementation Activities

In May 2009, the FMOH convened a consultative meeting with organizations involved in the health sector to discuss their roles in supporting the government's program to increase access to Implanon at the community level through capacity-building trainings, commodity and supplies support, and monitoring and evaluation of the program. At the FMOH's request, IFHP prepared a proposed implementation plan for the Implanon scale-up program, which was eventually validated and approved by the FMOH.

#### The Initial Learning Phase

The initial learning phase ran from July to September 2009 (Figure). The FMOH, in partnership with IFHP, selected 36 PHCUs from 8 *woredas* located in the 4 IFHP intervention regions of Amhara, Oromia, SNNPR, and Tigray to participate in the learning phase. These PHCUs were composed of 36 health centers and 192 health posts. IFHP and the FMOH organized 4 training-of-trainers (TOT) sessions in July 2009 for 72 clinical care providers—18 to 20 participants in each session—from the participating health centers. The TOT sessions included a total of 6 days of in-class theoretical training, practical sessions simulating Implanon insertion and removal practice on arm models, and, finally, supervised clinical practicums with clients. After successfully completing the session, the newly certified trainers conducted a series of rollout trainings with HEWs.

**FIGURE:**
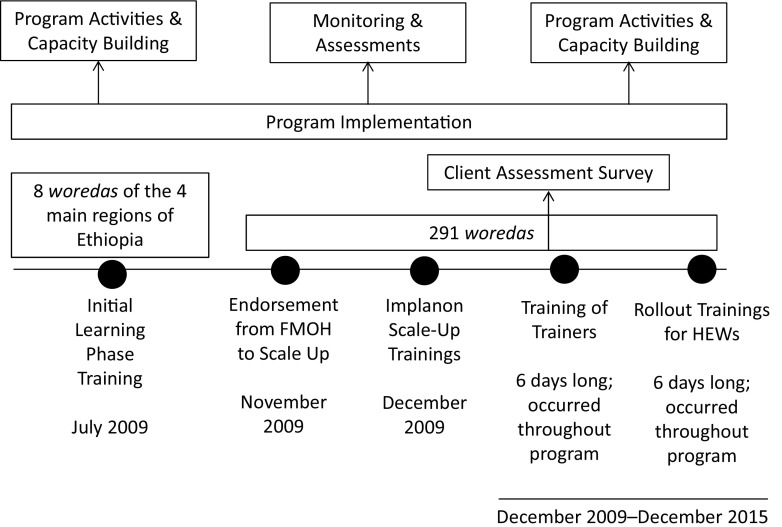
Implanon Scale-Up Program Components and Implementation Abbreviations: FMOH, Federal Ministry of Health; HEWs, Health Extension Workers.

**Figure:**
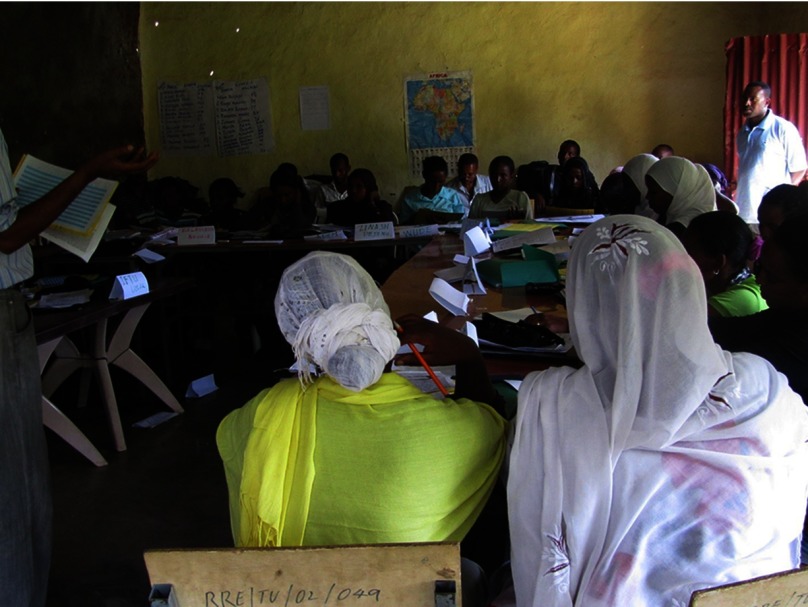
Health Extension Workers learn about Implanon during a theoretical training session. © Pathfinder International Ethiopia.

**Figure:**
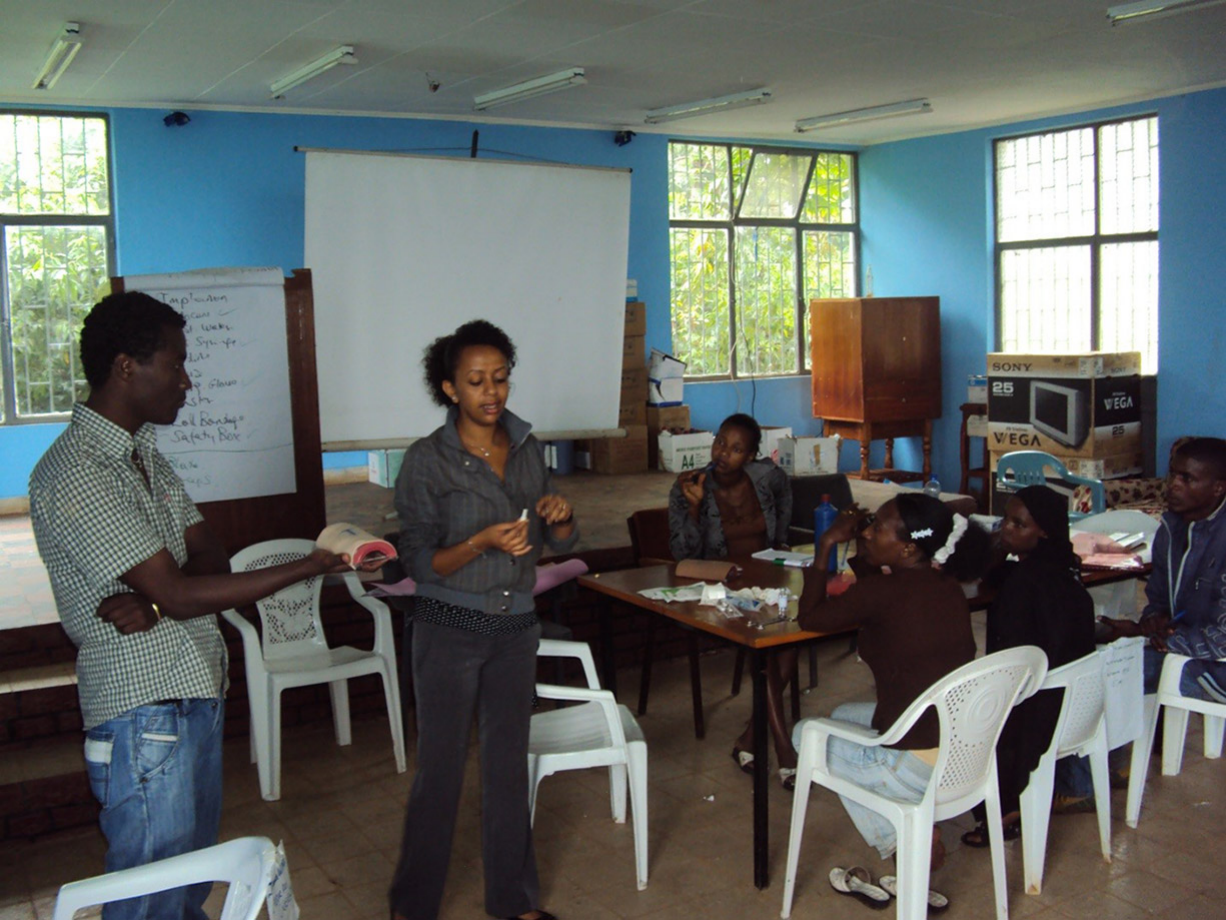
An instructor simulates Implanon insertion on an arm model to Health Extension Workers during a training session. © Pathfinder International Ethiopia.

**Figure:**
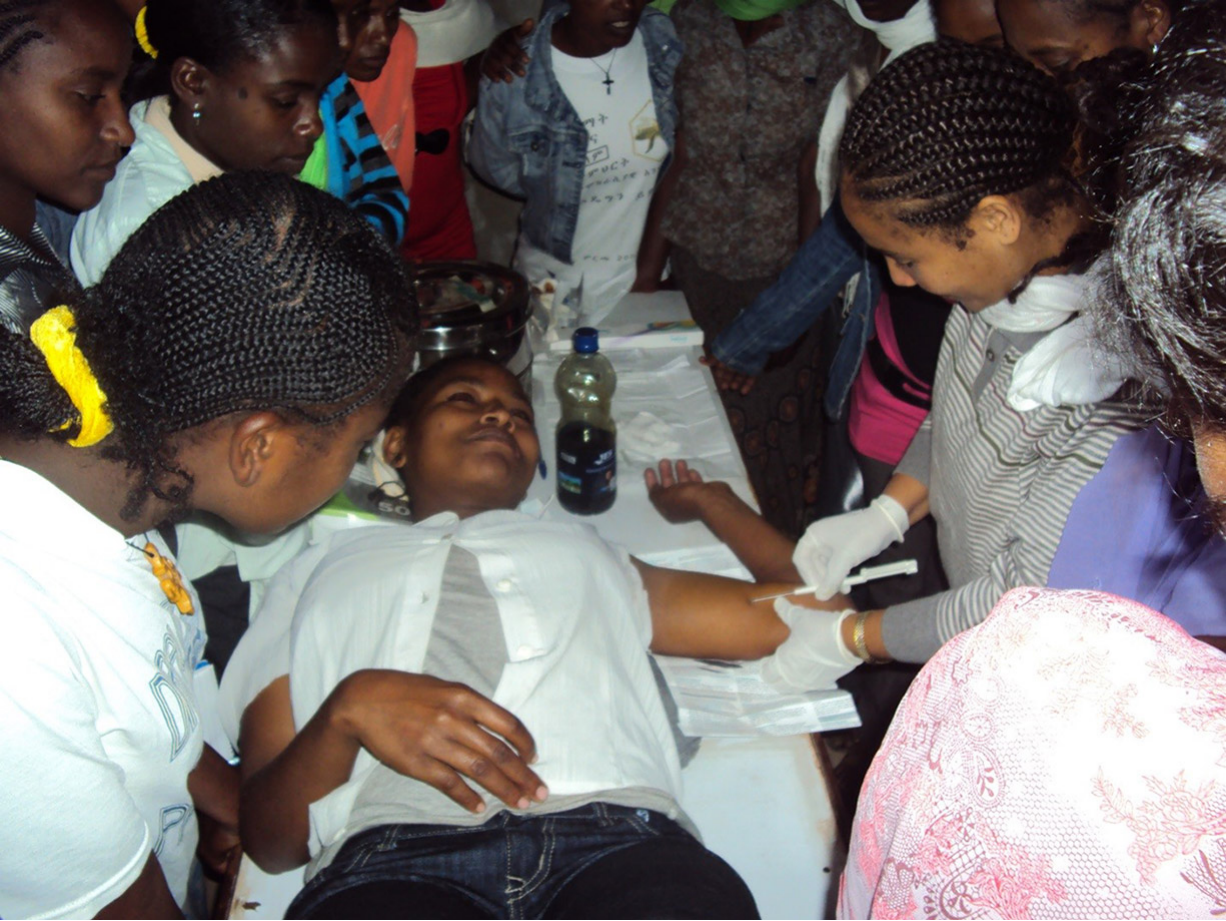
Under the supervision of an instructor, a Health Extension Worker inserts an Implanon implant in a client during training, as other trainees watch the procedure. © Pathfinder International Ethiopia.

During the learning phase, a total of 218 HEWs were trained on contraceptive counseling and Implanon insertion through a series of 9 rollout trainings, with an average of 25 HEWs in each rollout training session. Both the TOT and the rollout trainings were conducted in accordance with the national Implanon curriculum, as instructed by the FMOH. Recognizing the critical importance of comprehensive family planning counseling, this curriculum includes instruction on appropriate ways to counsel clients on all contraceptive methods, an essential component of ensuring informed consent and wide method choice, and proper pre-insertion Implanon counseling, which includes discussion of possible side effects and information about how to access Implanon removal services.

During the learning phase, a total of 218 HEWs were trained on contraceptive counseling and Implanon insertion through a series of 9 rollout trainings, with an average of 25 HEWs in each rollout training session.

HEWs were required to satisfactorily insert a minimum of 5 Implanon implants before they were considered competent. IFHP supported the FMOH by providing post-training commodities and supplies to trainees to allow them to immediately begin offering Implanon services at their respective health posts. To increase awareness and generate demand for the services, 2 weeks prior to the learning phase service days, IFHP deployed a mobile van with a speaker to inform the community about different family planning services, including Implanon, to be offered through the training sessions.

**Implanon removal services.** A critical element of rights-based contraceptive programming is implant removal services, yet, as data suggest, too often programs fail to adequately plan for equitable access to removals.^9^ Because HEWs are not permitted to remove Implanon in Ethiopia, IFHP supported the FMOH to implement the following 3 key strategies to ensure accessibility of removal services: (1) the program provided comprehensive implant removal training to 72 health care providers—doctors and nurses—stationed at health centers during the learning phase and an additional 2,328 health care providers from December 2009 to December 2015; (2) IFHP supported regular “back-up services,” wherein doctors and nurses from the health centers traveled to health posts at a prespecified time to offer removal services to clients at the community level; and (3) IFHP sent mobile teams to *woredas* that were outside of IFHP's catchment area to serve women in need of Implanon removal. Prior to 2010, implant removal services in Ethiopia were only available at a limited number of public health facilities and at a few clinics operated by NGOs such as Marie Stopes International. Details about these implant removal strategies will be explained in a separate publication.

**Evaluation of the learning phase.** Following the TOT and rollout trainings held during the learning phase, IFHP supported the FMOH to conduct post-training follow-up/mentorship visits to health posts at least twice during the 3-month follow-up period—September to December 2009. During follow-up visits, IFHP used a competency-based checklist to assess the HEWs' skills as well as general service provision at health posts, such as contraceptive counseling, infection prevention practices, and availability of commodities. As part of follow-up, HEWs collected information on the number of implants inserted; occurrence of side effects, defined as menstrual changes during the first 2 years of Implanon use, such as amenorrhea or no bleeding, infrequent or frequent bleeding or spotting, prolonged bleeding, headache, nausea, breast pain, and mood changes; complications; and requests for premature—within 3 to 6 months of insertion—removal of implants.

After 3 months of post-training follow-up, IFHP facilitated performance review meetings in each region with family planning experts; FMOH managers; regional, zonal, and district health officers; NGO representatives; trainers and trained HEWs; and health center heads. During these meetings, participants reviewed client service data, summary reports documenting follow-up and mentorship visits, client testimony on Implanon services provided by HEWs, and participant observations of counseling, insertion procedure skills, and infection prevention techniques during live demonstrations by HEWs.

#### Implanon Scale-Up Phase

In November 2009, the FMOH evaluated the results of the learning phase and began a nationwide scale-up of the program. During the scale-up phase—December 2009 to December 2015—IFHP, in partnership with the FMOH, conducted 98 TOT sessions with 2,328 clinical care providers and 320 rollout training sessions with 8,436 HEWs from 291 participating *woredas* within the IFHP program area. Both the TOT sessions and the rollout trainings for HEWs were facilitated using the same approach as that of the learning phase with 2 exceptions: (1) the clinical practicum session for HEWs was extended from 2 to 3 days to ensure that HEWs had sufficient time to practice their skills under supervision; and (2) the number of practicum sites and clinical trainers was increased to a minimum of 6 sites and 6 trainers for each training session—up from 3 to 4 during the learning phase—in order to ensure a sufficient level of client demand and HEW competency.

From December 2009 to December 2015, IFHP, in partnership with the FMOH, conducted 98 TOT sessions with 2,328 clinical care providers and 320 rollout training sessions with 8,436 HEWs from 291 participating *woredas* within the IFHP program area.

Furthermore, in September 2011, based on findings from a client survey conducted from December 2009 to June 2010 and performance review meetings, IFHP supported the FMOH to introduce a 3-part program support system to increase Implanon service access and coverage, including implant removals, as detailed below.

1. **Increase the amount of post-training consumables and commodities support provided to trainees:** IFHP increased the number of immediate post-training commodity and supply kits provided from 30 to 60 per HEW, in order to meet client demand and allow immediate initiation of services after training.
2. **Provide gap-filling of commodities and consumables:** To support uninterrupted continuation of services after the HEWs' post-training supplies and commodities were exhausted, IFHP filled commodity and consumable gaps during regular Implanon service follow-up and technical support visits through “gap-filling” support.
3. **Promote and support participation of the public health sector in the program:** In all of the 291 program-supported *woredas*, IFHP worked to transfer skills and experience to the districts to promote local ownership of the program through activities such as program planning, organization and facilitation of trainings, post-training follow up and mentoring, facilitation of review meetings, and use of client service data for decision making, thus ensuring sustainability of the program.

Although program activities during the learning phase were focused on the provision of Implanon by HEWs, all clients were provided with family planning counseling and offered the full method mix of contraceptive methods available during the Implanon trainings, and, subsequently received the method of their choice.

### Data Collection and Analysis

To assess programmatic performance and understand client needs, we collected and analyzed data from the Implanon scale-up phase, including the number of TOT and rollout trainings, number of providers trained, and number of clients served during the learning and scale-up phases through available service delivery points—health centers, health posts, and back-up family planning service support to health posts.

From December 2009 to June 2010, IFHP also conducted a series of client assessment surveys to identify areas for programmatic improvements and the need for Implanon services at the community level. All clients contacted by IFHP who sought an implant during the TOT sessions, rollout trainings, and clinical practicum period (N=7,254) agreed to participate in the assessment. (In our experience, we have found that clients in the IFHP catchment area are usually willing to provide information if they know that the assessment is to be used for program improvement). We used a structured questionnaire to collect client sociodemographic data, current and/or previous family planning use, and source of information about Implanon services. Health care providers asked clients the survey questions after counseling them and before providing their method of choice. We derived aggregate total service delivery indicators and basic descriptive statistics from 2 data sources: routine quantitative programmatic data and client assessment data.

## RESULTS

### Availability of Trained Providers

From July 2009 to December 2015, IFHP facilitated 98 TOT sessions with 2,328 clinical health care providers stationed at the health-center level. The program's subsequent 320 rollout trainings trained 8,436 HEWs on Implanon insertion, representing 73.5% of the total 11,476 HEWs in the IFHP area (Table 1). By December 2015, Implanon insertion services were available at 6,079 health posts—96.4% of all the health posts within the IFHP catchment areas (Table 1). The addition of back-up services from health centers to health posts resulted in a greater than anticipated number of acceptors of all methods during these support services.

**TABLE 1.** Number of HEWs Trained on Implanon Insertion and Number of Health Posts With Trained HEWs,^a^ by Region, July 2009 to December 2015

Name of Region	HEWs			Health Posts		
	Total No. of HEWs	No. of Trained HEWs	% of HEWs Trained	Total No. of Health Posts	No. of Health Posts With Trained HEWs	% of Health Posts With Trained HEWs
Amhara	3,885	2,204	56.7	1,891	1,891	100.0
Oromia	3,861	2,997	77.6	2,553	2,501	98.0
SNNPR	2,934	2,536	86.4	1,467	1,310	89.3
Tigray	796	699	87.8	398	377	94.7
**Total**	**11,476**	**8,436**	**73.5**	**6,309**	**6,079**	**96.4**

Abbreviations: HEW, health extension worker; SNNPR, Southern Nations, Nationalities, and People's Region.

a Includes health posts with at least 1 HEW trained on Implanon insertion.

### Use of Implanon Insertion and Removal Services

As shown in Table 2, from July 2009 to December 2015, a total of 82,702 service visits were made for family planning services through TOT sessions and rollouts. During the same period, an additional 1,181,000 women received Implanon insertion services from HEWs at the health-post level with implants supplied through IFHP's post-training and gap-filling commodity interventions. By ensuring sufficient supplies, the IFHP enabled HEWs to immediately initiate the service after training and thus continue to exercise their Implanon insertion skills. This support also increased use and coverage of family planning in the community.

**TABLE 2.** Contraceptive Services Provided to Clients by HEWs During and After Training, and by Nurses and Doctors through Back-Up and Outreach Services^a^

	No. (%) of Service Visits Served by HEWs			No. (%) of Service Visits Served by Other Health Care Providers			Total No. (%) of Service Visits
	During Training Sessions^b^	With Post-Training Supplies Provided to HEWs^c^	With Gap-Filling Supply Support to Health Posts After Post-Training Supplies Exhausted^c^	During Back-Up Services^d^	During Implant Removal Service Support at Health Centers^e^	During Outreach Implant Removal Service Support^f^	
	Jul 2009 to Dec 2015	Jul 2009 to Dec 2015	Sep 2011 to Dec 2015	Sep 2011 to Dec 2015	Sep 2011 to Aug 2012	Sep 2011 to Aug 2012	Jul 2009 to Dec 2015
**Total service visits**	**82,702 (100.0)**	**446,010**	**735,000**	**89,177 (100.0)**	**20,498 (100.0)**	**8,931 (100.0)**	**1,382,318 (100.0)**
**LARC insertions**	**67,662 (81.8)**	**446,010**	**735,000**	**35,414 (39.7)**			**1284086 (92.9)**
Implanon insertions	63,989 (77.4)	446,010	735,000	28,991 (32.5)			1,273,990 (92.2)
Jadelle insertions	3,590 (4.3)			4,321 (4.8)			7,911 (0.6)
IUD insertions	83 (0.1)			2,102 (2.4)			2,185 (0.2)
**Short-acting methods**	**12,320 (14.9)**			**32,716 (36.7)**			**45036 (3.3)**
Depo-Provera injectables	10,214 (12.4)			25,231 (28.3)			35,445 (2.6)
Oral contraceptive pills^g^	2,106 (2.5)			4,950 (5.6)			7,056 (0.5)
Condoms				2,535 (2.8)			2,535 (0.2)
**LARC removals**	**2,720 (3.3)**			**21,047 (23.6)**	**20,498 (100.0)**	**8,931 (100.0)**	**53,196 (3.8)**
Implanon removals	1,163 (1.4)			17,302 (19.4)	14,389 (70.2)	4,321 (48.4)	37,175 (2.7)
Jadelle removals	191 (0.2)			1,461 (1.6)	2,637 (12.9)	686 (7.7)	4,975 (0.4)
IUD removals	23 (0.03)			75 (0.1)			98 (0.01)
Norplant removals	1,343 (1.6)			2,209 (2.5)	3,472 (16.9)	3,924 (43.9)	10,948 (0.8)

Abbreviations: HEW, Health Extension Worker; IFHP, Integrated Family Health Program; IUD, intrauterine device; LARC, long-acting reversible contraceptive; TOT, training of trainers.

a Data for the different program activities in the table were collected at different points during the program period; this table does not include insertions performed by doctors and nurses at the health center level.

b Includes both TOT and rollout training sessions.

c Post-training supplies were provided to HEWs to allow them to provide immediate services and gap-filling supply support was provided to health posts after post-training supplies were exhausted. HEWs also provided other contraceptive services to clients post-training, but these data are not included in this table.

d IFHP supported regular back-up services, whereby doctors and nurses from health centers traveled to health posts to offer removal services for LARC clients.

e In addition to doctors and nurses traveling from the health centers to health posts to offer removal services, the doctors and nurses also provided removal services at the health centers.

f IFHP mobile teams traveled to *woredas* outside the project catchment area to serve women in need of implant removals.

g Includes both combined oral contraceptive pills and progestin-only pills.

Over 6 years, nearly 83,000 service visits were made for family planning services through TOT sessions and rollouts, and an additional 1.2 million women received Implanon from HEWs at health posts.

A further 89,177 service visits were provided through the back-up resource (Table 2). Out of which 28,991 (32.5%) service visits were for Implanon insertion, another 39,139 (43.9%) received other contraceptive methods. Nearly 20% (17,302) of back-up clients received Implanon removal services and about 4% (3,745) received removal of other LARC methods. Anecdotal evidence from health care provider observations, suggests that the back-up strategy likely positively affected client satisfaction, as they no longer needed to travel long distances to health centers to receive their method of choice.

By strengthening health-center level static implant removal services, 20,498 implant removals—70.2% Implanon, 12% Jadelle, and 16.9% Norplant—were provided to clients between September 2011 and August 2012. Finally, a total of 8,931 women living outside the IFHP catchment area had their implants removed through support provided to other *woredas*. Throughout the data collection period, IFHP supported 53,196 service visits made for implant removal services (Table 2).

### Method Mix

As mentioned earlier, by December 2015, 6,079 (96.4%) IFHP-supported health posts had added Implanon insertion services by HEWs to their existing family planning method mix (Table 1). In addition, regularly scheduled back-up services from health centers to health posts were designed to provide community access to implant removal services. Table 2 illustrates that in addition to removals, these back-up services provided a significant number of clients with both long- and short-acting methods, demonstrating access to and acceptance of a wider method mix than previously available at the community level. These back-up services provided contraceptive methods for an additional 4,321 Jadelle service visits, 2,102 IUD service visits, 25,231 Depo-Provera service visits, 4,950 oral contraceptive pill service visits, and 2,535 condom service visits (Table 2).

In addition to removals, back-up services provided a significant number of clients with both long- and short-acting methods, demonstrating access to and acceptance of a wider method mix than previously available at the community level.

### Client Assessment

As shown in Table 3, of the 7,254 clients seeking an implant from December 2009 to June 2010, 25.3% (1,837) were new family planning acceptors who had never used contraception before. An additional 63.6% of clients switched from Depo-Provera and 6.3% switched from pills, illustrating shifts from short- to long-acting methods. The majority (86.4%) of respondents received Implanon insertion, and the remaining clients chose either the Jadelle insertion (12.7%) or other family planning methods (0.9%) (Table 3).

**TABLE 3.** Sociodemographic Characteristics of Implant Clients, December 2009 to June 2010 (N=7,254)^a^

Characteristics	Frequency	Percentage
**Age, years**		
<24	1582	21.9
25–29	2254	31.2
30–34	1850	25.6
35–39	1097	15.2
≥40	448	6.2
**Place of residence**		
Urban	531	7.3
Rural	6720	92.7
**Educational status**		
Illiterate	6442	88.8
Literate	812	11.2
**Parity**		
1	712	10.2
2	999	14.4
3–6	4108	59
≥7	1138	16.4
**Previous use of family planning**		
New user (no previous method)	1837	25.3
Oral contraceptive pills	454	6.3
Depo-Provera injectables	4616	63.6
Other method	344	4.8
**Current method**		
Implanon implants	6221	86.4
Jadelle implants	916	12.7
Other method	61	0.9
**Length of use, years**		
<1	2959	57.5
1–3	1668	32.4
4–5	389	7.6
≥6	133	2.6
**Source of information about implant**		
Health Extension Worker	4786	71.1
Health worker	457	6.8
Volunteer community health worker	884	13.1
Other	609	9.1

a Totals for each variable do not always equal 7,254 because some respondents did not answer all questions. Percentages are calculated based on the total number of respondents for each individual question.

The client assessment survey also revealed areas for programmatic improvement. First, effective demand creation resulted in a significant need for LARC services at the community level that was not being met by HEWs. Second, a shortage of resources limited the capacity of health posts to meet client needs—even for other family planning services routinely provided by the HEWs at the health-post level. Third, demand for implant removal services at the community level increased. Last, we saw a greater demand for a range of family planning services not normally provided at the health-post level by HEWs, such as IUDs and Jadelle insertion and removals.

## DISCUSSION

In low- and middle-income countries where resource shortages and health-system weaknesses complicate service delivery, community health workers can play a critical role in expanding access to contraception. Through task sharing, the contraceptive method mix available at the community level can be increased; however, task sharing is often challenging to execute.^10^ Several programs have succeeded at improving access to contraception at the community level by engaging community health workers to deliver a range of methods, most notably injectables.^11^

This practice has been recognized as a USAID high-impact practice.^12^ Our experience contributes to this evidence by demonstrating the successful use of task sharing to increase access to LARCs at the community level in Ethiopia.

We discovered a larger-than-anticipated unmet need for Implanon in our program areas. One in 4 Implanon acceptors in our client assessment survey was a new family planning acceptor. This is in line with the findings of a 2013 survey, which discovered that 23.1% of Implanon acceptors during training events in the 4 IFHP regions were new family planning users.^13^ This evidence suggests that when implant insertion services are brought closer to the community, those with a latent need are able to seek out and demand these services.

1 in 4 Implanon acceptors in our client assessment survey was a new family planning acceptor.

Moreover, by training providers at the health-center level to remove implants, initiating back-up services, and supporting non-IFHP affiliated *woredas* with removals, the program supported the FMOH to increase access to removal services for women in settings that prohibit lower-level health workers from performing the service. As shown in Table 2, IFHP saw a substantial number of women seeking LARC removal services at both the health centers (38.5% of total removals) and back-up services at health posts (32.5% of total removals).

The FMOH's ability to scale the program is also noteworthy. Implementers often face substantial challenges when scaling up a successful pilot program. As shown in Table 2, IFHP achieved considerable scale through the Implanon scale-up program. From July 2009 to December 2015, a total of 1,273,990 Implanons were inserted in the IFHP areas.

Further illustrating scale, the IFHP endline survey—conducted in 2013—showed that the modern contraceptive prevalence rate (CPR) had substantially increased from baseline (27.4% in 2008 to 2009) to endline (39.1% in 2013) in the 4 program regions.^14^ The baseline survey indicated implant prevalence was at 0.7%; by 2013, this prevalence had increased to 6.3%, due in large part to the Implanon scale-up program.^13^ Moreover, the Ethiopia 2015 contraceptive commodity and service assessment showed that implant availability among HEWs and health posts exceeded 70%, mostly due to availability of Implanon.^15^ By 2013, almost 75% of all health posts provided Implanon insertion services. This report also showed that across all methods and cadres, 20% of total couple-years of protection came from HEW and health post implant insertions.^14^

In 2013, the IFHP endline survey showed that the modern CPR had substantially increased from baseline (27.4% in 2008 to 2009) to endline (39.1% in 2013) in the 4 program regions.

From the beginning of the Implanon scale-up initiative, the FMOH owned the Implanon scale-up program and IFHP provided support to launch the program and increase service utilization at the community level. This created an enabling environment, which filtered down to the district level and facilitated program ownership and sustainability. The FMOH led the program design and implementation processes, and held a consultative meeting with regional- and district-level health office experts. Representatives from these levels also actively participated in trainings and conducted post-training follow-up mentoring. The program design allowed the regional health managers to organize and conduct the post-training performance review meetings in their respective *woredas.* Each *woreda* also benefits from having a standing pool of trainers from the public sector, which was organized through the TOT trainings in the Implanon scale-up program. These trainers are able to quickly organize and conduct Implanon trainings. Public-sector ownership is evidenced by the regional health bureaus funding back-up support to health posts—about 60 PHCUs in Oromia and 10 PHCUs in SNNPR have done this so far.

Finally, our experience shows that the TOT and rollout-training approaches appear to have been effective. Almost all the HEWs proved to be competent at Implanon insertion immediately following the skills training. Their skills were further reinforced through mentoring. Providing HEWs with commodities and supplies immediately following the training was an important strategy that made it possible for the immediate and sustained quality provision of implants at the community level.

### Limitations

Our study design is subject to several limitations. First, because our study was descriptive in nature, we were unable to determine whether alternative strategies, such as training HEWs on Implanon in a preservice setting, may have achieved the same results as our strategies. Further, the client assessment was conducted only at the beginning of the program. We were unable to repeat the client assessment toward the end of the program, which would have allowed us to determine whether client profiles had changed over time. We would recommend that future studies explore client satisfaction, particularly with regard to which service setting is preferable to clients. Finally, from a programmatic sustainability perspective, despite good engagement with the public sector throughout the Implanon task-sharing initiative, allocation of public-sector resources to the new service has been slow.

## CONCLUSION

The Implanon scale-up program in Ethiopia is a successful model of increasing access to LARC methods in the community. Scale up was possible through task sharing of LARC services by training frontline health providers, specifically HEWs, and establishing program support interventions to address identified gaps. The program demonstrated unmet demand for LARCs in the community and showed that access to quality services can be improved with interventions, such as task sharing, regional and local health system support, and a strong commodity supply system.

## Notes

**Peer Reviewed**

**First Published Online:** December 11, 2017

**Funding:** USAID.
